# Unveiling the rare coexistence: thyroid hemiagenesis and thyroid cancer - case series and comprehensive review

**DOI:** 10.3389/fendo.2026.1841194

**Published:** 2026-05-14

**Authors:** Guiming Fu, Hongxia Zhou, Ting Wan, Yinfeng Li, Zhaohui Wang, Yanjia Hou

**Affiliations:** Department of Thyroid-ENT Head & Neck Surgery, Sichuan Clinical Research Center for Cancer, Sichuan Cancer Hospital & Institute, Sichuan Cancer Center, University of Electronic Science and Technology of China, Chengdu, China

**Keywords:** clinical characteristics, epidemiology, surgery, thyroid cancer, thyroid hemiagenesis

## Abstract

**Background:**

Thyroid hemiagenesis (THA) is a rare congenital malformation. By contrast, the co-occurrence of thyroid carcinoma (TC) in patients with THA is even rarer.

**Case reports:**

We present a detailed account of the diagnosis and treatment of two patients diagnosed with THA complicated by TC.

**Materials and methods:**

we performed a comprehensive search of the *PubMed* in accordance with the *PRISMA 2020* guidelines. Subsequently, we performed a detailed summary and analysis of the diagnostic and therapeutic data of the included cases.

**Results:**

We identified 32 cases of THA complicated with TC reported in the past 50 years. 5 were male and 27 were female. 24 patients underwent color Doppler ultrasonography (US), 12 received CT scanning, and 19 underwent fine-needle aspiration biopsy (FNAB). A total of 29 patients were treated surgically. Papillary thyroid carcinoma (PTC) accounted for 81.3% of all enrolled cases.

**Conclusions:**

The coexistence of THA and TC is exceedingly rare and might potentially be linked to multiple genes. There remains a paucity of data on the recognized incidence and etiology of this condition. Currently, diagnostic approaches to this disease are largely analogous to those for conventional TC. Surgical resection remains the primary treatment for such patients; however, the determination of resection scope remains controversial. It is recommended that surgical strategies be formulated with reference to the clinical guidelines for conventional TC. To date, there have been no comprehensive reports evaluating the surgical complications and long−term therapeutic outcomes of this disease.

## 1 Introduction

Thyroid hemiagenesis (THA) is a rare congenital malformation characterized by normal development of one thyroid lobe and absence of the contralateral lobe, with or without the presence of the isthmus. Its incidence in the general population is less than 0.1%, and it is more common in females and on the left side (1, 2). Since most patients have normal thyroid function and no clinical symptoms, THA is usually accidentally detected during neck ultrasound (US) or imaging examinations performed for other thyroid diseases (3). Papillary thyroid carcinoma (PTC) is the most common endocrine malignancy, whose typical ultrasonic features include hypoechogenicity, ill-defined borders, an aspect ratio greater than 1, and microcalcifications. When PTC occurs in patients with THA, due to anatomical variations, preoperative imaging evaluation is prone to misjudge the absent side as “postoperative changes” or “atrophy”, thereby leading to deviations in the decision of surgical scope, such as unnecessary expansion of the resection range to total thyroidectomy (4). At present, case reports of THA combined with PTC are extremely rare, and there is no unified consensus on its clinical diagnosis and treatment strategy, especially in young female patients, relevant reports are even scarcer. In this article, we describe in detail the diagnostic and therapeutic approaches taken with two asymptomatic THA + PTC young female patients encountered in recent years. Additionally, we summarize the diagnostic and therapeutic details of previously reported cases discovered through a comprehensive literature review, aiming to provide valuable insights for the management of similar cases in the future.

## 2 Case reports

### 2.1 Case 1

A 27-year-old female patient was referred to our hospital for further evaluation and treatment. Two months prior, during a routine physical examination at a local hospital, a low-echo nodule measuring approximately 19×7 mm was incidentally detected in the right lobe of the thyroid gland via color Doppler US. The nodule was categorized as American College of Radiology Thyroid Imaging Reporting and Data System (ACR TI-RADS) 4B. No left thyroid lobe or isthmus was detected. Subsequently, under the guidance of color Doppler US, the patient underwent fine-needle aspiration biopsy (FNAB), which confirmed the diagnosis of PTC. Given the findings, the patient exhibited significant anxiety and was subsequently referred to our hospital by the local medical team for comprehensive assessment and surgical management.

**Figure 1:**
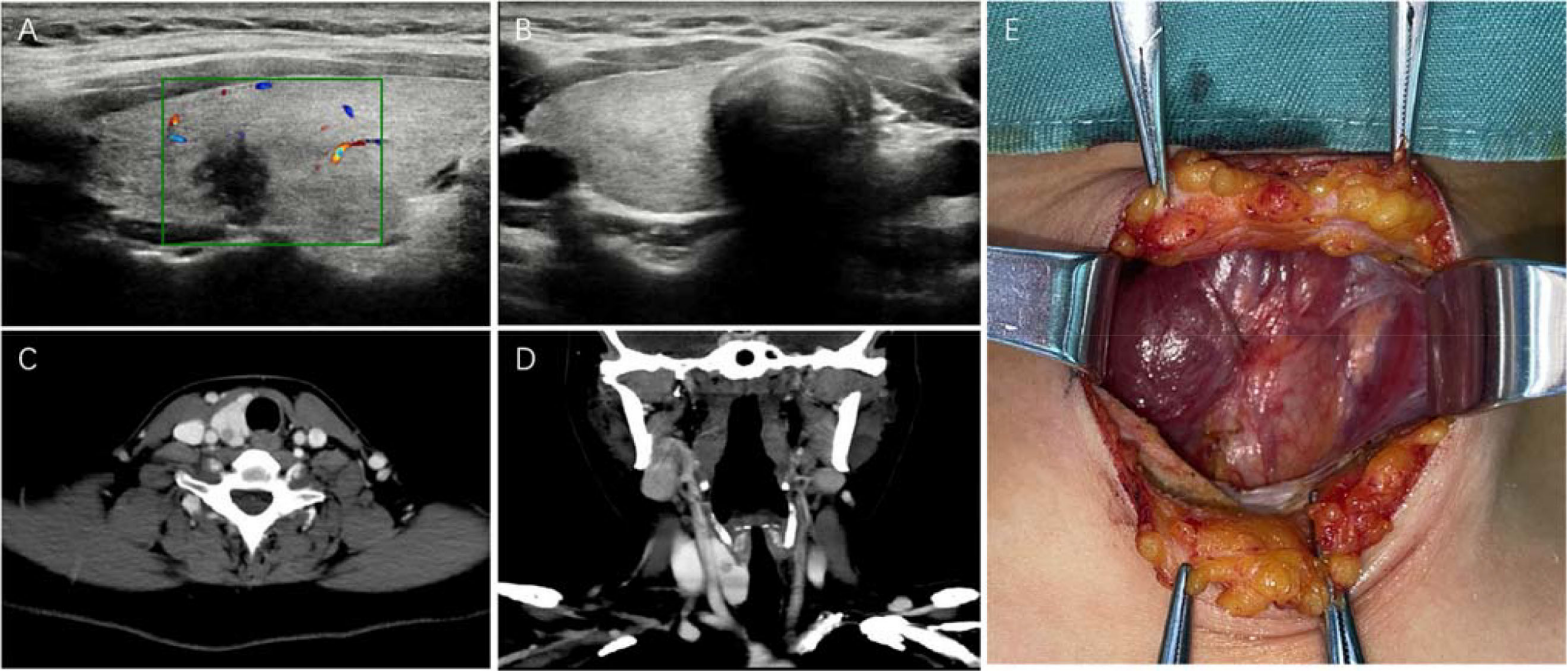
Case 1: **(A)** illustrates the US appearance of a nodule in the right lobe of the thyroid gland using color Doppler imaging. **(B)** demonstrates the absence of the left lobe and isthmus of the thyroid gland as identified by color Doppler US. **(C–D)** depict the CT findings of the right lobe of the thyroid gland and the associated nodule. **(E)** confirms the absence of the left lobe and isthmus of the thyroid gland during surgical exploration.

On admission, no palpable nodules were detected in the right lobe of the thyroid, and no enlarged lymph nodes were observed bilaterally in the neck. Preoperative thyroid function tests were all within normal limits. Color Doppler US of the neck revealed two hypoechoic nodules with extremely low echogenicity in the mid-portion of the right thyroid lobe, one measuring 15×7×6 mm and the other 2×3×3 mm. Both exhibited unclear margins, irregular shapes, and a longitudinal growth pattern with a longitudinal-to-transverse ratio > 1. The nodules were classified as ACR TI-RADS grade 6 (**Figure 1A**). Additionally, multiple lymph nodes were identified in the right cervical VI region on color Doppler US, some showing structural abnormalities. No left thyroid lobe or isthmus was detected. (**Figure 1B**). Enhanced neck CT confirmed the presence of small nodules in the right thyroid lobe and the absence of the left lobe and isthmus (**Figures 1C, D**). No enlarged lymph nodes were noted in the neck. Other investigations, including chest CT, laryngoscopy, and electrocardiogram, revealed no significant abnormalities. The patient had no remarkable medical history or family history of genetic disorders.

In response to the patient’s significant anxiety, we performed open-neck surgery in December 2022. The operation involved resection of the right thyroid lobe and prophylactic dissection of the right-central cervical lymph nodes. Intraoperatively, we confirmed that the left thyroid lobe and isthmus were absent (**Figure 1E**), with no parathyroid glands (PTGs) identified in the left-lobe region. Both PTGs on the right side were successfully preserved. Postoperatively, the patient recovered well and was discharged without complications such as hoarseness, dysphagia, or paresthesia in the hands and feet. Final pathological paraffin sections revealed two PTC lesions in the right lobe of the thyroid gland (one measuring 5 mm and the other 1 mm in diameter). Additionally, a *BRAF-V600E* gene mutation was detected. Metastasis was identified in one out of four right-central cervical lymph nodes. The postoperative pathological stage of the patient was pT1N1aM0, stage I. Following surgery, the patient received levothyroxine replacement therapy, with thyroid function maintained within normal limits. She has been followed up on for 40 months without evidence of tumor recurrence or metastasis.

### 2.2 Case 2

**Figure 2:**
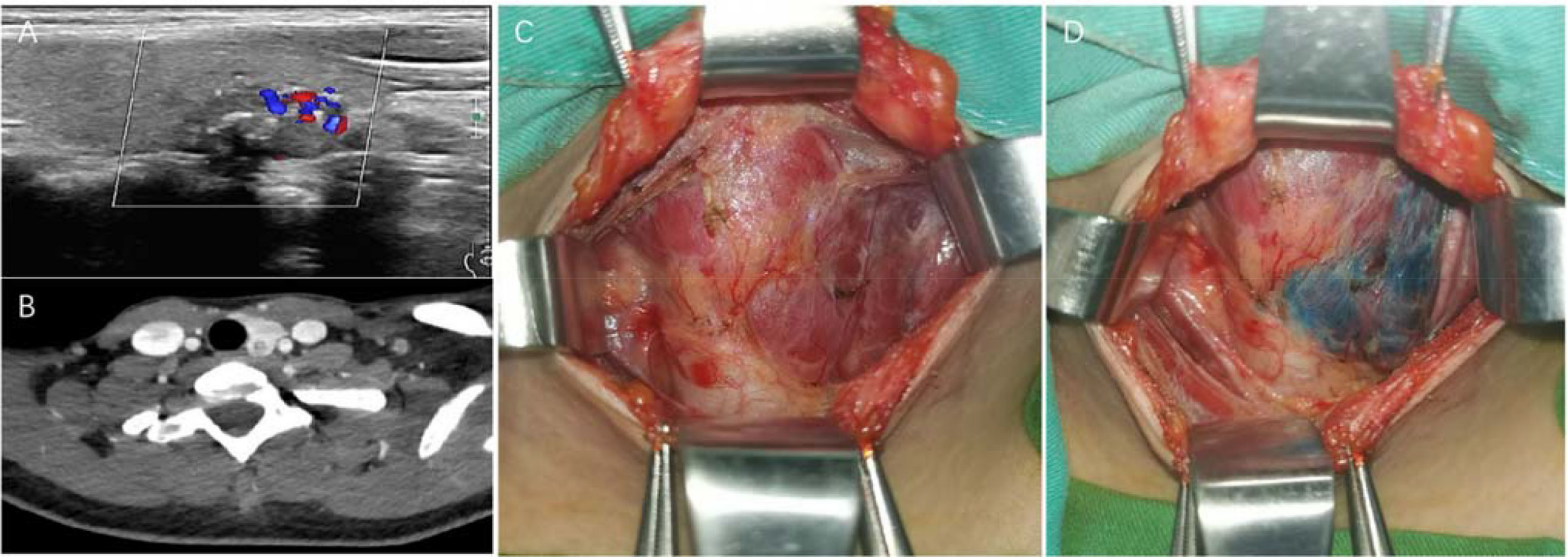
Case 2: **(A)** depicts the US characteristics of a nodule in the left lobe of the thyroid gland using color Doppler imaging. **(B)** presents the CT findings of the left lobe of the thyroid gland and the nodule, with evidence of the absence of the right lobe and isthmus of the thyroid. **(C–D)** illustrate the intraoperative appearance of the left lobe thyroid gland before **(C)** and after **(D)** thyroid staining.

A 23-year-old female patient was identified during a routine physical examination. Color Doppler US revealed a low-echo nodule in the middle and lower portions of the left thyroid lobe, measuring approximately 18×11× 11 mm, with unclear boundaries, irregular shape, and scattered punctate calcifications (**Figure 2A**). Additionally, the boundary between this nodule and the posterior capsule of the thyroid gland was indistinct. The nodule was categorized as ACR TI-RADS 4B. Multiple lymph nodes were detected in the VI region of the left neck, some of which exhibited structural abnormalities. Notably, color Doppler US did not detect the presence of the right lobe or isthmus of the thyroid gland. Given her family history of TC, the patient declined FNAB under color Doppler US guidance and requested direct surgical excision.

On physical examination, no palpable nodules were noted in the left thyroid lobe, and no significant lymphadenopathy was observed bilaterally in the neck. Preoperative thyroid function test results were all within normal limits. Enhanced neck CT confirmed the presence of a small nodule in the left thyroid lobe and the absence of the right lobe and isthmus (**Figure 2B**). Results of all other preoperative investigations were unremarkable.

Given the tumor invasion of the posterior thyroid capsule as demonstrated on color Doppler US and CT imaging, we recommended traditional open-neck surgery for the patient. On March 19, 2025, we performed a left-thyroid lobectomy and central lymph node dissection on the left side of the neck. Intraoperatively, we confirmed that the right lobe and isthmus of the thyroid were absent, and the nodule in the left lobe had invaded the posterior thyroid capsule (**Figures 2C, D**). Both PTGs on the left side were successfully preserved. Intraoperative frozen-section pathology confirmed PTC with posterior-capsule involvement in the left-lobe nodule. Postoperatively, the patient recovered well, with normal levels of parathyroid hormone and calcium on re-examination. She was discharged 3 days later without complications such as hoarseness or dysphagia. Final paraffin section pathology revealed metastasis in two out of six lymph nodes in the left-central neck region, and a *BRAF-V600E* gene mutation was detected. The patient’s postoperative pathological stage was pT1N1aM0 (Stage I). She subsequently received levothyroxine replacement therapy and has completed her the third follow-up examination, with all laboratory results within normal limits.

## 3 Materials and methods

Given the extreme rarity of concurrent TC and thyroid THA, we subsequently performed a comprehensive search of the *PubMed* database in accordance with the *PRISMA 2020* guidelines. Both Medical Subject Headings (MeSH) and free-text terms were used for the search. The search terms included:Thyroid hemiagenesis, thyroid dysgenesis, thyroid agenesis, unilateral thyroid agenesis, thyroid aplasia, and congenital thyroid hypoplasia. The search process was conducted independently and in parallel by two investigators. Any discrepancies were resolved through discussion or adjudication by a third investigator to ensure the reproducibility and objectivity of the search process. The search was conducted from database inception to 2026. Subsequently, we performed a detailed summary and analysis of the diagnostic and therapeutic data of the included cases.

## 4 Results

After full-text review and screening of the retrieved articles, we identified only 32 cases of THA complicated with TC reported in the past 50 years. The detailed diagnosis and treatment of the 32 previously reported cases are summarized in **Table 1**. Among the 32 patients, 5 were male and 27 were female, with a male-to-female ratio of 1:5.4. Nineteen cases manifested left thyroid lobe agenesis, 10 exhibited right lobe agenesis, and 3 presented with agenesis limited to the isthmus. At least 22 patients had normal thyroid function, accounting for no less than 68.8% of all cases. Five patients were diagnosed before the age of 30, 23 were aged 30 to 60 years, and 4 were 60 years or older. 50.0% of patients had concomitant primary thyroid comorbidities. Color Doppler US was performed in 24 cases, CT in 12, FNAB in 19, Tc−99m scintigraphy in 9, ¹³¹I examination in 4, and MRI in 1.A total of 29 patients received surgical treatment. Of these, approximately 78.1% underwent thyroid lobectomy or lobectomy combined with isthmectomy, while only 31.3% received concurrent cervical lymph node dissection. Postoperative pathological findings indicated that PTC was the most common histological subtype, accounting for 81.3% of all cases.

**Table 1:** A systematic review of the literature from 1970 to the present reveals data on patients with THA + TC.

No.	Study	Ref	Year	Sex	Age	Missing lobe of thyroid	Isthmus of thyroid	Diagnostic method	Thyroid function	Extent of resection	Iodine 131 therapy	Type of thyroid cancer	Other diseases
1	Hamburger, J. I., et al	(11)	1970	F	14	Left	/	^131^I	/	Right lobe	/	PTC&FTC	/
2	Harada T, et al	(12)	1972	F	74	Right	Exist	^131^I	Normal	Subtotal left lobe & Modified radical left neck dissection	No	PTC	/
3	Letonturier, P, et al	(13)	1979	F	52	Left	/	Tc-99m	Normal	Right lobe	/	PTC	/
4	Greening, W. P., et al	(14)	1980	F	51	Left	No	/	/	The nodule in the right lobe	No	PTC	Tuberous goiter
5	Khatri, V. P., et al	(15)	1992	F	41	Right	No	^131^I	Normal	Left lobe	/	PTC	/
6	McHenry CR, et al	(16)	1995	F	58	Left	Exist	/	/	/	/	FTC	/
7	Ashok R. Shaha, et al	(17)	1997	F	30	Right	No	US&^131^I&FNAB	/	Left lobe	/	PTC	/
8	Shih-Ming Huang, et al	(18)	2002	F	47	Right	Exist	US&CT&Tc-99m&FNAB	Normal	Left lobe, isthmus, and pyramidal lobe	No	PTC	Ectopic prelaryngeal thyroid gland
9	Pizzini, A. M., et al	(19)	2005	M	54	Left	No	US&Tc-99m& FNAB	Normal	The right lobe and isthmus	Yes	PTC	Tuberous goiter
10	Ammaturo C, et al	(20)	2007	F	39	Left	/	CT	/	Left lobe	/	PTC	Flajani's disease
11	Francesco Berni Canani, et al	(21)	2008	F	35	Left	No	US&MRI	Normal	The tumor in the midline of the neck	No	PTC	Thyroglossal cyst
12	Yong Sang Lee, et al	(22)	2008	F	69	Left	Exist	US&CT&FNAB	/	Right lobe & Central cervical lymph node	No	PTC	Thyroid adenoma
13	Yusuf Vayisoglu, et al	(23)	2013	F	43	Isthmus	No	US& FNAB	Normal	Total thyroidectomy & Central cervical lymph node	/	PTC	/
14	Gülden Yenice Karatağ, et al	(24)	2013	F	59	Left	Exist	US&Tc-99m&FNAB	Normal	Right lobe	No	PTC	Adenomatous hyperplasia
15	Wang J, et al	(24)	2014	F	49	Right	No	US&Tc-99m	Normal	Left lobe & Left central lymph node & Left cervical lymph nodes	No	MTC	/
16	Wang J, et al	(25)	2014	F	60	Left	Exist	US&Tc-99m & FNAB	Normal	The right lobe and isthmus & Right central lymph node	Yes	PTC	Tuberous goiter &lymphocytic thyroiditis
17	Alfredo Campennì, et al	(26)	2015	M	36	Left	No	US&Tc-99m&FNAB	Hyperthyroidism	Total thyroidectomy&Cervical lymph nodes	Yes	PTC	Graves' disease
18	Sakorafas, G. H., et al	(27)	2015	F	47	Left	/	US& FNAB	/	/	/	PTC	/
19	Rajbhandari P, et al	(28)	2016	M	28	Isthmus	No	US& FNAB	Normal	Total thyroidectomy	/	PTC	Lymphocytic thyroiditis
20	Hiroki Sato, et al	(29)	2017	F	64	Left	Exist	US&CT&FNAB	Normal	The right lobe and isthmus+Tumor in the left paratracheal trachea & Central cervical lymph node	Yes	PTC&Poorly differentiated carcinoma	/
21	Ugur K, et al	(30)	2019	F	54	Isthmus	No	US&FNAB	Normal	Total thyroidectomy & The ectopic thyroid mass	No	PTC	Ectopic thyroid cancer
22	Sowjanya Gandla, et al	(31)	2020	F	20	Right	Exist	US&CT&FNAB	Normal	Left lobe	No	PTC	/
23	Saad M. Alqahtani, et al	(32)	2021	F	36	Right	Exist	US&CT&Tc-99m&FNAB	Normal	Left lobe & Left lower parathyroid gland	No	PTC	Parathyroid adenoma&lymphocytic thyroiditis &Tuberous goiter
24	Saad M. Alqahtani, et al	(32)	2021	M	40	Left	Exist	US& FNAB	Normal	The right lobe and isthmus	No	PTC	lymphocytic thyroiditis
25	Diani Kartini, et al	(33)	2021	F	59	Right	Exist	US& FNAB	Normal	The left lobe and isthmus	No	Oncocytic Carcinoma	/
26	Shruti Dhingra, et al	(34)	2022	F	24	Left	No	US&CT&Tc-99m&FNAB	Normal	Modification of Sistrunk's procedure	/	PTC	Thyroglossal cyst
27	Yanko G. Yanko, et al	(35)	2023	F	14	Left	Exist	US&CT	Normal	The tumor in the midline of the neck	No	PTC	Thyroglossal cyst
28	Kexin Meng, et al	(36)	2023	F	46	Right	Exist	US&CT	/	The left lobe and isthmus&Left central lymph node	No	PTC	/
29	Dae Young Yoon, et al	(37)	2023	F	52	Left	/	CT	Hypothyroidism	/	/	PTC	Hypothyroidism
30	Lee CH, et al	(38)	2024	F	37	Left	Exist	US&FNAB	Normal	The left lobe and isthmus	Yes	PTC	/
31	Li C, et al	(39)	2025	M	33	Right	Exist	US&CT&FNAB	Normal	Total thyroidectomy,&bilateral central lymph node & left cervical compartment	No	MTC	/
32	Chen S, et al	(4)	2026	F	36	Left	No	US&CT	Normal	Right lobe & Central cervical lymph node	No	PTC	/

Sex, F/female, M/male; US, ultrasound; CT, computed tomography; MRI, magnetic resonance imaging; FNAB, fine-needle aspiration biopsy; PTC, papillary thyroid carcinoma; FTC, follicular thyroid carcinoma.

## 5 Discussion

### 5.1 Epidemiology

The development of the thyroid gland begins during the fourth week of embryogenesis. Initially, the endodermal epithelial cells located between the ventral ends of the first and second pharyngeal arches proliferate and invaginate to form the thyroid diverticulum, which subsequently elongates into the thyroglossal duct. The thyroglossal duct traverses the tongue and extends downward anterior to the hyoid bone and thyroid cartilage, forming a cellular cord. As this cord descends, it reaches the level of the second to fourth tracheal rings, where the upper portion undergoes regression, while the lower portion expands and grows laterally to eventually form the bilateral lobes and isthmus of the thyroid gland (5). In 1842, Todd RB was the first to describe an anatomical variation in which one lobe of the thyroid gland develops normally, while the contralateral lobe is absent (6).

As a rare anomaly of congenital developmental, THA is typically asymptomatic, and its true prevalence in the general population remains unclear. A large meta-analysis conducted by Mikosch P et al. in 1999 estimated the prevalence of THA to be approximately 0.05%, with a male-to-female ratio of 1:4.33 and a left-to-right ratio of 3.6:1. The absence of the isthmus accounted for 44% of all cases of hypoplasia (7). A Belgian study reported that among 2845 healthy children who underwent US screening, the prevalence of THA was 0.2%, with a male-to-female ratio of 1:2, and all cases involved left-lobe agenesis (8). An US-based study involving 24,032 children age 11–14 years found a prevalence of 0.05%, with a male-to-female ratio of 1:1.4, and the majority of cases exhibited left-lobe agenesis (9). Gursoy A et al. conducted a survey of 4833 patients with thyroid disorders and 7707 healthy individuals; the results showed THA to have a prevalence of 0.25% in patients with thyroid disease and 0.025% in the general population, with a male-to-female ratio of 1:3. Nearly all cases demonstrated left-lobe agenesis (10).

By systematically reviewing the literature on PubMed, we identified approximately 300 published case reports on THA. Based on these findings, we estimated an overall incidence in the population of approximately 1:10,000, accounting for 15–20% of all congenital thyroid developmental abnormalities. The occurrence of TC is even more infrequent in patients with THA than in those with THA alone. We estimated that the incidence of TC in THA patients accounted for approximately 8–10% of all THA cases, representing roughly 1 in 100,000 individuals in the general population. The current data indicated that women were significantly more predisposed to THA than men. Women also predominated among THA + TC patients, constituting 84.4% of the 32 reported cases.

### 5.2 Pathogenesis

The rarity of THA has long limited our understanding of its exact pathogenesis. However, accumulating evidence indicates that this condition might be associated with genetic alterations involved in the transcriptional regulation of thyroid development and the control of thyroid–mesenchymal migration during human embryogenesis. To date, mutations to such genes as thyroid transcription factor-1 (*TTF1*), forkhead box protein E1 (*FOXE1*), paired box protein 8 (*PAX8*), and natural killer 2 homeobox 5 (*NKX2-5*); microduplications in the 22q11.2 chromosomal region harboring the T-box transcription factor 1 (*TBX1*) gene; and variations in homeobox proteins B3 and D3 (*HOXB3*, *HOXD3*), paired-like homeodomain transcription factor 2 (*PITX2*), glioma-associated oncogene homolog 1 (*GLI3*), and proteasome genes have been identified in THA patients. These genetic changes are suspected to contribute to the pathogenesis of THA.

Previous gene knockout studies in mice have demonstrated that *TTF1* and *PAX8* are critical to the survival and proliferation of thyroid follicular-cell precursors during thyroid development (40–42). Additionally, *TTF2* is essential to the downward migration of these cells (43), while the thyrotropin receptor plays a key role in postnatal thyroid growth (44). In 2011, Szczepanek E et al. conducted a study of 40 THA patients and found that the incidence of long *FOXE1* polyalanine (*FOXE1*-polyAla) tract variants was significantly higher in familial cases than in sporadic cases and controls. This suggests that *FOXE1*-polyAla tract expansion might be associated with the molecular basis of familial THA (45). In 2013, Kim HJ et al. were the first to report the detection of 22q11.2 microduplications in the amniotic fluid of a fetus with congenital hypothyroidism and THA, indicating that congenital thyroid dysplasia should be considered in patients with 22q11.2 microduplications (46). It is well established that the *HOX* gene family is expressed during early embryogenesis and involved in thyroid development, particularly *HOXA3*, which has a closer association with thyroid formation. *PITX2* is responsible for determining left–right patterning and the positioning and symmetry of internal organs. In 2014, Kizys MM et al. investigated mutations to *HOXA3*, *HOXB3*, *HOXD3*, and *PITX2* in four THA patients. The results revealed no significant sequence variations or potentially harmful mutations in these genes, suggesting that other candidate genes might underlie thyroid asymmetry in humans (47).

In 2017, Budny B et al. performed genomic analyses of 34 sporadic cases of THA and one three-generation family. The results identified four recurrent defects affecting highly conserved proteasome genes, including proteasome 20S subunits α1, α3, and δ3 (*PSMA1*, *PSMA3*, *PSMD3*). In a thyroid hemiagenesis family a splice site mutation in a proteasome gene *PSMD2* (c.612T > C cDNA.1170T > C,g.3271T > C) was found in both affected mother and daughter. This study was the first to demonstrate that genomic alterations in protein-related genes could contribute to this developmental anomaly through proteasome mutations (48). Subsequently, Li M et al. identified three putatively pathogenic *PAX8* mutations in 289 patients with thyroid dysgenesis (TD) for the first time, revealing a *PAX8* mutation rate of 1.04% in Chinese patients with TD and thereby expanding the known mutational spectrum associated with this condition (49). In 2020, Szczepanek-Parulska E et al. established a unique association between THA phenotype and compound heterozygous mutations *p.[Gly727Arg];[Gln1347Lys]* to *GLI3* in two siblings with unilateral thyroid agenesis (50). Furthermore, genetic variations in *NKX2–5* were subsequently identified in siblings with THA, providing novel insights into the pathogenesis of this form of TD (51).

The aforementioned studies have elucidated multiple potential genetic causes underlying THA. Furthermore, researchers have recognized more and more that the development of THA is unlikely to be governed by a single gene but rather involves the concerted regulation of multiple genes. This raises important questions: Is THA hereditary? Are individuals with THA at higher risk of developing TC? Based on current evidence, these two conditions appear to represent rare independent events that happen to co-occur in some cases. However, this hypothesis warrants further investigation in future research.

### 5.3 Clinical features and treatment

THA is often asymptomatic, and most patients are incidentally diagnosed (1). In contrast, patients with THA + TC are frequently identified due to the presence of other thyroid disorders (4). Our presented data revealed that 50.0% of patients with THA combined with TC had concomitant primary thyroid comorbidities. This might be attributed to elevated thyroid-stimulating hormone (TSH) levels; chronic stimulation of the thyroid gland can lead to compensatory enlargement, inflammation, and even malignancy (52–54). Diagnostic approaches to THA + TC are similar to those for conventional TC, including color Doppler US, CT, and FNAB. Notably, our research revealed that prior to 2015, approximately 60% of THA + TC diagnoses were made using technetium-99m and iodine-131 scans. However, over the past decade, the utilization rate of these methods has decreased to about 14.3%. We consider that this shift is closely associated with advancements in the sensitivity of color Doppler US equipment and the increasing application of US-guided FNAB in thyroid nodule diagnosis. Clinicians might increasingly favor less radiation-intensive examination methods for patient safety. THA patients frequently exhibit compensatory enlargement of the remaining thyroid tissue. Thyroid function test results are normal in most cases, and therefore the majority of these patients do not require pharmacological or surgical intervention (55). Of the THA + TC patients we analyzed, at least 68.8% demonstrated normal thyroid function.

Based on our summary, surgical resection remains the primary treatment modality for THA + TC patients. Although 29 out of 32 reported cases (90.6%) underwent surgical treatment, the extent of resection varied significantly. Approximately 78.1% of these patients underwent thyroid lobectomy or lobectomy + isthmusectomy, while only 31.3% underwent concurrent cervical lymph node dissection. Postoperative pathological findings indicated that all types of TC can occur in this population, with PTC the most prevalent subtype, accounting for approximately 81.3% of occurrences. Intriguingly, we also observed that the proportion of patients undergoing cervical lymph node dissection was highest among those treated surgically between 2008 and 2017, reaching approximately 60%. It appears that the intensity of surgical treatment for these patients has undergone three phases: from minimal to maximal and back to minimal intensity. This trend is closely linked to the evolving understanding of TC within the medical community.

However, there are still several notable limitations in the present study. First, the necessity of prophylactic cervical lymph node dissection for such patients remains unclear. Although cervical lymph node metastasis was confirmed in both cases enrolled in this article, it is still uncertain whether routine prophylactic cervical lymph node dissection is required for other analogous patients. Second, available reports rarely describe surgical complications and disease prognosis in patients with THA+TC, and the relevant conclusions lack support from large−sample data and long−term follow−up evidence. Third, the overall number of currently reported cases is extremely limited. Therefore, multicenter cohort studies should be conducted in the future to systematically evaluate the biological characteristics and optimal surgical extent of THA+TC patients, so as to establish more evidence−based consensus on diagnosis and treatment.

### 5.4 Conclusions

The co-occurrence of THA and TC is exceedingly rare and might potentially be linked to multiple genes. There remains a paucity of data on the recognized incidence and etiology of this condition. Currently, diagnostic approaches to this disease are largely analogous to those for conventional TC, primarily depending on neck color Doppler US, neck CT, FNAB, or supplementary detection of *BRAF-V600E* gene mutations for auxiliary diagnosis. Surgical resection remains the primary treatment for such patients; however, the determination of resection scope remains controversial. It is recommended that surgical strategies be formulated with reference to the clinical guidelines for conventional TC. To date, there have been no comprehensive reports evaluating the surgical complications and long−term therapeutic outcomes of this disease.
